# Clinical Implications of *MiR128*, *Angiotensin I Converting Enzyme* and *Vascular Endothelial Growth Factor* Gene Abnormalities and Their Association with T2D

**DOI:** 10.3390/cimb43030130

**Published:** 2021-11-02

**Authors:** Imadeldin Elfaki, Rashid Mir, Faisel M. Abu Duhier, Maeidh A. Alotaibi, Adel Ibrahim Alalawy, Jameel Barnawi, Abdullatif Taha Babakr, Mohammad Muzaffar Mir, Faris Altayeb, Hyder Mirghani, Ehab A. M. Frah

**Affiliations:** 1 Department of Biochemistry, Faculty of Science, University of Tabuk, Tabuk 71491, Saudi Arabia; aalalawy@ut.edu.sa; 2 Prince and Fahd Bin Sultan Research Chair, Department of Medical Lab Technology, Faculty of Applied Medical Sciences, University of Tabuk, Tabuk 71491, Saudi Arabia; rashid@ut.edu.sa (R.M.); fabu-duhier@ut.edu.sa (F.M.A.D.); jbarnawi@ut.edu.sa (J.B.); F.Tayeb@ut.edu.sa (F.A.); 3 King Faisal Medical Complex Laboratory, Ministry of Health, Taif 26521, Saudi Arabia; maed96@hotmail.com; 4 Department of Medical Biochemistry, Faculty of Medicine, Umm Al-Qura University, Makkah 57039, Saudi Arabia; abdullatiftaha@yahoo.com; 5 Department of Basic Medical Sciences, College of Medicine, University of Bisha, Bisha 61992, Saudi Arabia; mirmuzaffar11@gmail.com; 6 Internal Medicine and Endocrine, Medical Department, Faculty of Medicine, University of Tabuk, Tabuk 71491, Saudi Arabia; h.mirghani@ut.edu.sa; 7 Department of Statistics, Faculty of Science, University of Tabuk, Tabuk 71491, Saudi Arabia; efrah@ut.edu.sa

**Keywords:** type 2 diabetes mellitus (T2D), genome wide association studies (GWAS), vascular endothelial growth factor (VEGF), *VEGF* insertion and deletion (I/D), *VEGF* rs699947, *ACE* I/D rs4646994, *Mir128a* (rs11888095)

## Abstract

Type 2 DM (T2D) results from the interaction of the genetic and environmental risk factors. Vascular endothelial growth factor (VEGF), angiotensin I-converting enzyme (ACE), and MicroRNAs (MiRNAs) are involved in important physiological processes. Gene variations in *VEGF*, *ACE* and *MiRNA* genes are associated with diseases. In this study we investigated the associations of the *VEGF*-2578 C/A (rs699947), *VEGF*-2549 insertion/deletion (I/D), and *ACE* I/D rs4646994 and *Mir128a* (rs11888095) gene variations with T2D using the amplification refractory mutation system PCR (ARMS-PCR) and mutation specific PCR (MSP). We screened 122 T2D cases and 126 healthy controls (HCs) for the rs699947, and 133 T2D cases and 133 HCs for the *VEGF* I/D polymorphism. For the ACE I/D we screened 152 cases and 150 HCs, and we screened 129 cases and 112 HCs for the *Mir128a* (rs11888095). The results showed that the CA genotype of the VEGF rs699947 and D allele of the *VEGF* I/D polymorphisms were associated with T2D with OR =2.01, *p*-value = 0.011, and OR = 2.42, *p*-value = 0.010, respectively. The result indicated the D allele of the *ACE* ID was protective against T2D with OR = 0.10, *p*-value = 0.0001, whereas the TC genotype and the T allele of the *Mir128a* (rs11888095) were associated with increased risk to T2D with OR = 3.16, *p*-value = 0.0001, and OR = 1.68, *p*-value = 0.01, respectively. We conclude that the *VEGF* (rs699947), *VEGF* I/D and *Mir128a* (rs11888095) are potential risk loci for T2D, and that the D allele of the *ACE* ID polymorphism may be protective against T2D. These results help in identification and stratification for the individuals that at risk for T2D. However, future well-designed studies in different populations and with larger sample sizes are required. Moreover, studies to examine the effects of these polymorphisms on VEGF and ACE proteins are recommended.

## 1. Introduction

Chronic diseases have often serious impact on the patients, their families and societies. Choosing an appropriate clinical investigation methodology is always essential to decide on proper treatment for patients [1,2]. Diabetes mellitus was one of the leading global causes of death in 2017 [3]. According to the WHO, Kingdom Saudi Arabia (KSA) ranks second in terms of DM prevalence and it has been estimated that more than 20% of Saudi population are diabetic [4]. This rate may be one of the highest in the world [4]. DM has very serious complications and consequences, such as diabetic retinopathy, diabetic nephropathy, diabetic neuropathy, cardiovascular diseases and amputation. DM is a metabolic disorder characterized by hyperglycemia. The insufficient insulin secretion by the pancreatic beta cells leads to the development of type 1 DM (T1DM) [5]. Type 2 diabetes (T2DM) results from the combination of insulin resistance initiated in major tissues, excessive hepatic production of glucose and pancreatic beta cells’ dysfunction [6]. T2DM represents more than 90% of all cases of DM [7]. T2D is induced by complex interactions of genetic and environmental risk factors [8]. Genome wide association studies (GWAS) have uncovered the association of certain loci with metabolic diseases including T2D [9,10,11,12,13,14,15]. Environmental risk factors of T2D include obesity, physical inactivity and unhealthy diet [7]. The vascular endothelial growth factor (VEGF) is a growth factor expressed in endothelial cells and is involved in angiogenesis [16]. The *VEGF* promoter gene variations (−2578C/A and −1154G/A) have been associated with metabolic syndrome in the Korean population [17]. Moreover, the VEGF rs10738760 (A/G) SNP was associated with metabolic syndrome in Iranian and Lebanese populations [18,19]. Angiotensin I-converting enzyme (ACE) is a nonspecific peptidase with multiple peptide substrates [20]. The ACE converts the angiotensin-I into angiotensin-II [20]. This conversion leads to the activation of the angiotensin-I peptide hormone into the vasoconstrictor angiotensin-II [21]. ACE is involved in important physiologic processes such as blood pressure, development and function of the kidney, reproduction, and the formation of blood cellular components [20]. The long-term inhibition of the ACE has been suggested as one of the preventive measures against T2D [22]. Furthermore, the *ACE* gene variations were associated with the progression of carotid artery disease in Slovenian T2D patients [23]. MicroRNAs are short noncoding RNA molecules that are involved in the regulation of gene expression [24,25]. It has been reported that there are elevated circulatory levels of miR-128 in patients with T2D and depression [26]. Moreover, the miR-128a rs11888095 was reported to be associated with diabetic neuropathy in the Italian population. In the present study we examined the association of *VEGF* rs699947 C/A (-2578), *VEGF*-2549 insertion/deletion (I/D), *ACE* I/D rs4646994 and *Mir128a* (rs11888095) gene variations with T2D in subjects from KSA.

## 2. Material Methods

### 2.1. Study Population, Inclusion and Exclusion Criteria

This project was approved by the Research and Studies Department, Directorate of Health Affairs, Taif, approval No. 229, and by the Research Ethics Committee of the Armed Forces hospitals, Northwestern Region approval No. R & RE C2016-115. The population comprised T2D patients vising the hospitals for routine checkup. The study included patients with clinically confirmed cases of T2D. The study included only citizens of Saudi Arabia, both males and females. All subjects gave informed consent. A standard questionnaire was used to document the socio-demographical characteristics such as age, sex and lifestyle. 

**Figure 1:**
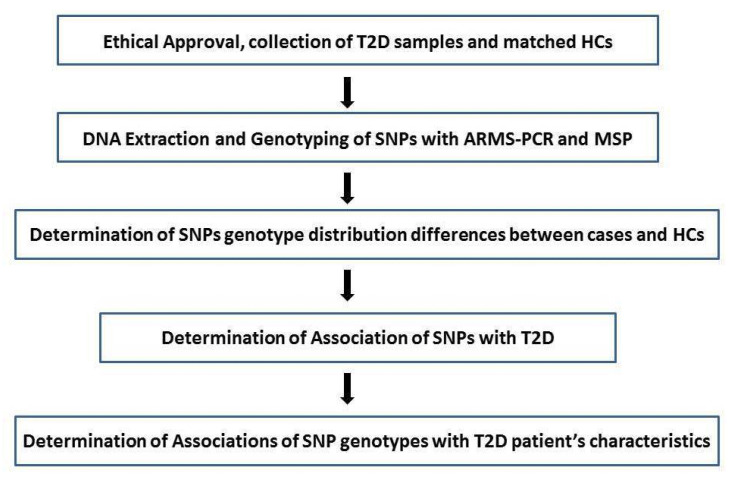
Summary of the study explaining the procedure from sample collection until statistical analyses. Abbreviations. HCs: healthy controls. ARMS-PCR: amplification mutation system PCR. MSP: mutation specific PCR.

The study excluded patients with T1D and patients with any previous history of any chronic diseases. The healthy controls (HCs) ranged from 20 to 80 years of age, and were visiting the hospital for a routine checkup. The controls were enrolled from the general population of the same geographical region. A routine medical check-up was conducted (CBC, KFT, LFT, etc.) and the history of illness, if detected, was recorded by a health practitioner. Those who appeared apparently healthy without any history of any significant disease, or other chronic diseases, were considered normal. Figure 1 summarizes the procedure of study from samples collection to the statistical analyses. 

### 2.2. Sample Collection and Genomic DNA Extraction

All patient specimens were timed around the routine drawing of blood that was the part of a routine workout, and hence did not require additional phlebotomy. About 3 mL of peripheral blood was collected by venipuncture in EDTA tubes from T2D patients and from HCs. DNA was extracted using a DNeasy Blood Kit (Cat No. 69506) Qiagen (Hilden, Germany) as per the manufacturer’s instructions, then the DNA was dissolved in nuclease-free water and stored at 4 °C until use. The extracted DNA was dissolved in nuclease-free H_2_O and stored at 4 °C until use. The quality of the extracted DNA was checked by running the sample in 0.8% agarose gel. The quantity of the extracted DNA was determined by NanoDrop™ (Thermo Scientific, Waltham, MA, USA).

### 2.3. Genotyping of VEGF rs699947C/A and miR-128a rs11888095 C/T by Amplification Refractory Mutation System PCR (ARMS-PCR)

VEGF promoter region rs699947 C/A and *mirR-128* rs11888095 C > T genotyping was conducted by the ARMS-PCR [27,28]. The primers were designed using Primer3 software (Table 1). The ARMS-PCR was performed in a reaction volume of 25 µL containing template DNA (50 ng), Fo-0.25 µL, Ro-0.25 µL, FI-0.25 µL and RI-0.25 µL of 25 pmol of each primer, and 10 µL from GoTaq Green PCR Master Mix (2X) (Promega, Madison, WI, USA). The final volume of 25 µL was adjusted by adding nuclease-free ddH2O. Then, 2 µL of DNA was added from each subject.

#### 2.3.1. ARMS-PCR Programming

The PCR conditions optimized for *VEGF* (rs699947C/A) and *miR128* rs11888095 C/T were with an initial denaturation at 95 °C for 10 min followed by 40 cycles of denaturation at 95 °C for 35 s; annealing Tm was 58 °C for *VEGF* rs699947 and 55 °C for *miR128a* rs11888095 for 40 s, extension at 72 °C for 45 s, and a final extension step at 72 °C for 10 min.

#### 2.3.2. Gel Electrophoresis for ARMS-PCR Products

**Figure 2:**
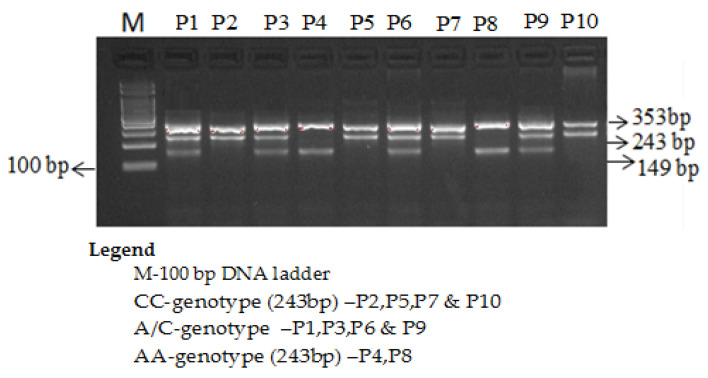
Genotyping of the *VEGF* rs699947 C/A using ARMS-PCR in T2D subjects.

The *VEGF* rs699947 (−2578 C/A) amplification products were separated by electrophoresis. Primers Fo and Ro flank the exon of the *VEGF*-2578 C/A gene, resulting in a band of 353 bp to act as a control for DNA quality and quantity. Primers FI and Ro amplify a wild-type allele (C allele), generating a band of 229 bp, and primers Fo and RI generate a band of 149 bp from the mutant allele (A allele) Figure 2.

**Figure 3:**
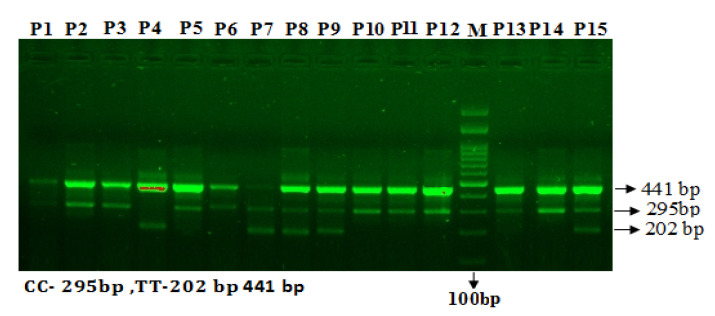
Genotyping of *miR128* rs11888095 C/T using ARMS-PCR in T2D patients. Legend: M—100 bp DNA ladder; Heterozygous C/T—P7, P8, P9 & P15; Homozygous CC—P1, P2, P3, P5, P6, P10, P11, P12, P13, P14; Homozygous TT—P4.

The *mir1*28 rs11888095 C/T amplification products were separated by electrophoresis. Primers Fo and Ro of *mir128* rs11888095 C/T resulted in a band of 458 bp to act as a control for DNA quality and quantity. Primers Fo and Ro amplify a wild-type allele (T allele), generating a band of 202 bp, and primers Fo and RI generate a band of 295 bp from the mutant allele (A allele) Figure 3.

### 2.4. Genotyping of VEGF I/D and ACE I/D rs4646994 by Mutation Specific PCR (MSP)

*VEGF* I/D and *ACE* I/D rs4646994 polymorphisms were genotyped using the MSP. with primers used by Amle et al. [29] for *VEGF* D/I. The primers were designed using the Primer3 software (Table 1). The PCR was undertaken in a reaction volume of 25 µL containing template DNA (50 ng), F-0. 25µL and R-0. 25 µL of 25 pmol of each primer, and 10 µL from GoTaq^®^ Green Master Mix (cat no. M7122) (Promega, Madison, WI, USA). The final volume of 25 µL was adjusted by adding nuclease-free double distilled water (ddH_2_O).

#### 2.4.1. MSP Programming

The PCR conditions used were initial denaturation at 95 °C for 10 min followed by 40 cycles of 95 °C for 35 s, annealing Tm for *ACE* I/D (58 °C) for 40 s and for *VEGF* I/D (58.80 °C) for 1 min, 72 °C for 45 s followed by the final extension at 72 °C for 10 min.

#### 2.4.2. Gel Electrophoresis for MSP Products

**Figure 4:**
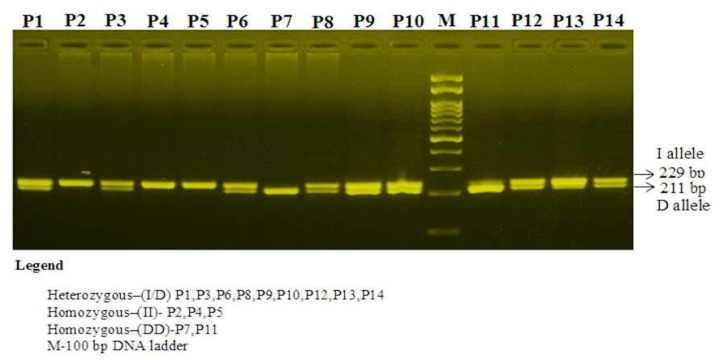
Mutation specific PCR for genotyping of *VEGF* I/D polymorphism in T2D subjects.

**Figure 5:**
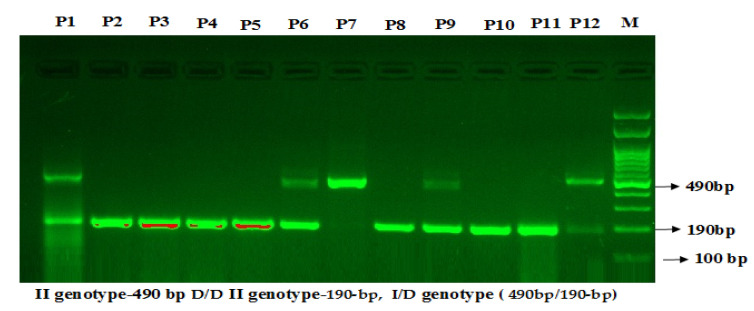
Genotyping of *ACE* I/D rs4646994 by mutation specific PCR in T2D. Legend: M—100 bp DNA ladder; Homozygous II genotype—P7; Homozygous DD genotype—P2, P3, P4, P5, P8, P10, P11; Heterozygous ID genotype—P1, P6, P9, P12.

The MSP of the product of the *VEGF*-2549 I/D products were separated on 1.5% agarose. There were two bands of 211 bp for the D allele and 229 bp for the I allele (Figure 4). The MSP product of *ACE*-I/D was separated on 1.5% agarose gel. There were 3 bands. The II genotype yielded a 490 bp fragment, the DD genotype yielded a 190 bp fragment, and ID yielded both 490 and 190 bp fragments (Figure 5).

### 2.5. Statistical Analysis

Group differences were compared using Student’s two-sample *t*-test or one-way analysis of variance (ANOVA) for continuous variables and chi-squared test for categorical variables. Deviations from the Hardy–Weinberg disequilibrium (HWD) were calculated by the chi-square (χ2) goodness-of-fit test. The differences in the *VEGF* rs699947 C/A, *VEGF*-2549 I/D, *miR128* rs11888095 C/T and *ACE* I/D rs4646994 genotype frequencies between cases and controls were evaluated using the chi-square test. Associations between alleles and genotypes and the incidence of T2D were estimated with the odds ratios (ORs), and the risk ratios (RRs). We calculated the risk differences (RDs) with 95% confidence intervals (CIs). A *p*-value < 0.05 suggested a significant difference. Statistical analyses were performed using Graph Pad Prism 6.0 or SPSS 16.

## 3. Results

### 3.1. Genotypes Distribution of the Gene Polymorphisms

Our results showed that the *VEGF* rs699947 C/A genotype distribution was significantly different between the cases (34.4, 57.4 and 8.2%) and the healthy controls (46, 38.1 and 15.9%) with a *p*-value = 0.007 (Table 2). The results also indicated that the distribution of the *VEGF I/D* polymorphism was significantly different between the cases (15, 53.4 and 31.6), and the controls (22.6, 57.9 and 19.5) with a *p*-value = 0.049 (Table 3). Moreover, results indicated that there was significant difference in the genotype distribution of the *ACE* I/D between the cases (57.2, 36.2 and 6.6%) and controls (12, 40 and 48%) with *p*-value = 0.049 (Table 4). The genotype distribution of the *mir128* rs11888095 C/T was also significantly different between cases (27, 53 and 20%) and the controls (55, 34 and 11%) with a *p*-value = 0.0001 (Table 5).

### 3.2. The Association of the VEGF rs699947 C/A SNP with T2D

The results indicated that *VEGF* rs699947 C/A SNP was associated with T2D, the CA genotype was associated with T2D with OR (95% CI) = 2.01 (1.17–3.45), RR = 1.42 (1.08–1.87), *p*-value = 0.011 (Table 6). Our results showed that there was a significant difference (*p*-value < 0.05) in the *VEGF* rs699947 C/A genotype distribution between male and female cases, and between cases >25 years and cases >40 years (Table 7). The results also showed that there were significant differences (*p*-values < 0.05) between different genotypes of the *VEGF* rs699947 in the patients with a normal lipid profile and cases with an abnormal lipid profile (Table 7).

### 3.3. The Association of VEGF-2549 I/D Polymorphism with T2D

Results indicated that the D allele of the *VEGF* I/D at the −2549 position was associated with T2D with OR (95% CI) = 1.43 (1.02–2.03), RR = 1.01 (0.83–1.21), *p*-value = 0.037 (Table 8). The results also showed that there were significant differences (*p*-values < 0.05) between different genotypes of the *VEGF*-2549 I/D polymorphism in patients with a normal lipid profile and patients with an abnormal lipid profile (Table 9).

The gender and age are based on data for 107 cases. HbA1c% is based on data for 97 cases, cholesterol mg/dl—82 cases, LDL-C mg/dl—85 cases, HDL-C mg/dl—38 cases, and vitamin D ng/mL (VIT.D)—37 cases.

### 3.4. Association of ACE I/D rs4646994 Polymorphism with T2D Patients

Results showed that the *ACE* I/D polymorphism was associated with T2D (Table 10). The ACE–ID genotype was associated with T2D with OR = 0.18 (0.101–0.354), RR = 0.32 (0.208–0.518), *p*-value = 0.0001 (Table 10). The *ACE*–DD genotype was also associated with T2D with OR = 0.128 (0.0125–0.0661), RR = 0.19(0.1272–0.2996), *p*-value = 0.0001 (Table 10). The D allele was associated with T2D with OR = 0.10 (0.12–0.2), RR = 0.40 (0.3–0.5), *p*-value = 0.0001 (Table 10). Our results indicated that there were a significant different in *ACE I/D* genotype distribution between cases aged >25 years and cases aged <25 years (Table 11). The results showed that there was a significant difference (*p*-value = 0.02) in the *ACE* I/D genotype distribution between cases with normal and cases with elevated HbA1c (Table 11). Moreover, results showed that there were significant differences (*p*-values < 0.05) in the *ACE* I/D genotype distribution in cases with normal and abnormal lipid profiles (Table 11).

### 3.5. Association of miR128 rs11888095 C/T SNP with T2D Patients

Results showed that the CT genotype of the *miR128* rs11888095 was associated with T2D with OR = 3.16 (1.8–5.6), RR = 1.78 (1.3–2.4), *p*-value = 0.0001 (Table 12). The results also indicated that the T allele was associated with T2D with OR = 1.68 (1.13–2.5), RR = 1.36 (1.0695–1.7400), *p*-value = 0.0105 (Table 12). Our results showed that there was a significant difference (*p*-value = 0.031) in the *miR128* rs11888095 genotype distribution between male and female cases (Table 13). Moreover, the results showed that there was a significant difference (*p*-value = 0.021) in cases aged <25 years and cases aged >25 years (Table 13). Moreover, results showed that there a significant difference (*p*-value = 0.0001) in cases with normal and cases with elevated HbA1c (Table 13). Furthermore, results showed that there was a significant difference (*p*-values < 0.05) in the genotype distribution of cases with normal and cases with elevated lipid profiles (Table 13).

## 4. Discussion

T2D is developed by a mixture of insulin resistance and impaired secretion of insulin [6]. The VEGF stimulates the cell proliferation, migration and vasopermeability in many tissues [30]. It has been reported that there is association between the VEGF expression and metabolic syndrome [31]. Metabolic syndrome is defined as insulin resistance associated with obesity, hypertension and dyslipidemia [31]. It has been suggested that increased levels of VEGF are associated with increased blood sugar [31]. Our results showed that there was a significant difference (*p*-value = 0.007) in the genotype distribution of *VEGF* rs699947 C/A between the cases and the controls (Table 2). The CA genotype was also associated with T2D (Table 6).

Results showed that there was a significant difference (*p*-values < 0.05) in the *VEGF* rs699947 genotype distribution between cases with normal and cases with abnormal lipid profiles (Table 7). This result is consistent with studies that reported the association of *VEGF* rs699947 with the risk of cardiovascular disease [32,33]. The results also showed that males with the rs699947 CA genotype and A allele are more susceptible to T2D than females (*p*-values < 0.05, Table 7). This result is consistent with studies reporting higher prevalence of T2D among males than females [34,35]. Our results also indicated that elder individuals (age > 40) with the CA genotype are more prone to T2D than younger individuals (<40 years old, *p*-values < 0.05, Table 6). This is also in agreement with a previous study that reported that T2D is more prevalent in older individuals in all populations [36]. We did not observe significant differences in the *VEGF* rs699947 genotype distribution in the cases with normal or elevated HBA1c (*p*-values > 0.05, Table 7). This is probably because of the relatively small sample size used in this study. This may be one of the limitations of this study.

The *VEGF* gene I/D polymorphism is an 18 bp fragment found at the −2549 position of the promoter region [29]. The results showed that there is a significant difference (*p*-value < 0.05) in the genotype distribution of *VEGF* I/D polymorphism between the cases and healthy controls (Table 3). The results indicated that the D allele of the *VEGF* I/D was associated with T2D (Table 8). Moreover, results indicated that there was a significant difference (*p*-values < 0.05) between genotypes of the *VEGF* I/D polymorphism in the patients with normal and patients with abnormal lipid profiles (Table 9). Gene variations in the *VEGF* gene promoter such as I/D polymorphism were reported to increase expression of the *VEGF* [30,37]. Therefore, these results may be in agreement with the result of Zafar et al., who reported that the increased expression of the VEGF gene has been associated with metabolic syndromes, such as hypertriglyceridemia [31]. Several previous studies have implicated the associations of diabetes complications with gene variations at the *VEGF* [38]. For instance, the *VEGF* gene at position −7 C/T was reported to be associated with diabetic neuropathy in a population of British Caucasians [39]. In addition, the −634 G/C SNP in the 5′UTR of the *VEGF* gene was associated with risk diabetic retinopathy (DR) in the Japanese population [40]. Furthermore, Buraczynska et al. showed that the *VEGF* I/D polymorphism was associated with risk of DR in the Polish population [37]. Churchill et al. reported the association of the *VEGF* promoter SNPs rs735286 and rs2146323 with the severity of DR in the British population [41], whereas Han et al. reported the association of *VEGF* gene SNPs rs3025039 and rs833061 with DR in the Chinese population [42]. Furthermore, the −2578 C/A, rs699947, was associated with diabetic foot ulcers in Iranian and Chinese Han populations [43,44]. All these studies may be in agreement with our results as they report the association of *VEGF* gene variations with diabetes complications, and our results showed that the *VEGF* gene variations rs699947 and *VEGF* I/D polymorphisms were associated with the risk of T2D (Table 6 and Table 8). Furthermore, it has been reported that the gene variations in the *VEGF* promoter (e.g., *VEGF* rs699947 and *VEGF* I/D) result in enhanced expression of the VEGF gene [30,37]. The increased expression of the VEGF gene was associated with insulin resistance [31], and that the neutralization of the VEGF gene resulted in improvement in insulin sensitivity in the liver and in fat tissues [45]. These studies may be in agreement with our results, which showed that *VEGF* rs699947 and *VEGF* I/D are associated with T2D risk.

Our results indicated that there was a significant difference in the *ACE* I/D polymorphism genotype distribution between cases and controls (*p*-value < 0.05, Table 4). The *ACE* I/D polymorphism was associated with T2D (Table 10). The results indicated that the *ACE* ID genotype and the *ACE* D allele were associated with decreased risk of T2D (Table 10). This result may be partially consistent with the results of Al-Serri et al. (2015) [46], who reported that the I allele of the *ACE* I/D polymorphism is associated with T2D in the Kuwaiti population. However, our result is in disagreement with the study of Al-Rubeaan et al. (2013) [47]. This disagreement may be due to different sample sizes or different populations. The results indicated that there was a significant difference in the *ACE I/D* polymorphism genotype distribution between males and females (Table 11). The results also showed that there were significant differences in the *ACE* I/D polymorphism genotype distribution between cases with elevated and cases with normal HbA1c, and between cases with normal and cases with abnormal lipid profiles (Table 11). These results are perhaps in agreement with studies reporting the association of *ACE* I/D polymorphism with dyslipidemia [48,49]. A previous study also proposed the ACE as a target for the protection of pancreatic beta cells from dysfunction causing T2D [50].

Results indicated the *miR128* rs11888095 may be associated with development of T2D (Table 5 and Table 12). The CT genotype and T allele of the *miR128* rs11888095 were associated with risk of T2D (Table 12). This result is consistent with a study reporting that miR128 regulates genes (e.g., *Insr*, *Irs1* and *Pik3r1*) critical for insulin signaling [51]. Our result is in partial agreement with a study indicating that the T allele of the *miR128* rs11888095 is associated with diabetic polyneuropathy [52,53]. The results showed that females carrying the CT genotype and T allele of the *miR128* rs11888095 are more susceptible to T2D than males carrying the CT genotype and T allele (Table 13). Furthermore, individuals with the CT genotype and T allele aged >25 years are more susceptible to T2D (Table 13). In addition, results showed that the CT genotype and T allele were more frequently present in cases with elevated HbA1c (>6%) than in cases with normal HbA1c (<6%) (Table 13). This result is expected because *miR128* rs11888095 may be associated with T2D (Table 12). Moreover, it was indicated that *miR128* rs11888095 may be associated with an elevated lipid profile (Table 13); this is quite consistent with a study reporting that mir128 regulates the gene involved in lipid metabolism [54]. Limitations of this study include the small sample size, and that it is a cross-sectional study, i.e., the samples may have been collected from cases after the blood chemistry was already maintained. Future longitudinal studies with larger sample sizes and in different populations are required. Because T2D can be delayed or prevented by diet modification, weight management, regular exercise and other factors [55,56], results of the present study can be used (after confirmation in future studies) in genetic testing and personalized advice to identify and stratify the individuals that at are risk of developing T2D.

## 5. Conclusions

We investigated the association of the *VEGF* promoter gene variations (*VEGF* rs699947, *VEGF* I/D), *ACE* I/D (rs4646994) and *miR128* (rs11888095) with T2D in the Saudi population. The results showed that the CA genotype of the *VEGF* rs699947, the D allele of the *VEGF* I/D, and the TC genotype and T allele of the *Mir128a* (rs11888095) were associated with an increased risk of T2D. Moreover, the results indicated the D allele of the *ACE* I/D was protective against T2D. Further well-designed studies with larger sample sizes in different populations are required. Moreover, proteomics investigations [57,58,59] to examine the effects of SNPs on VEGF and ACE function are recommended.

## Figures and Tables

**Table 1:** Genotyping primers of gene polymorphisms.

Primers for VEGF-rs699947 (−2578) C/A Gene Polymorphism		Band	Band Size	A.Tm
VEGF Fo	5-CCTTTTCCTCATAAGGGCCTTAG-3	Control band	353 bp	58 °C
VEGF Ro	5-AGGAAGCAGCTTGGAAAAATTC-3			
VEGF FI A	5-TAGGCCAGACCCTGGCAA-3	A-allele	149bp	
VEGF RI C	5-GTCTGATTATCCACCCAGATCG-3	C-allele	243bp	
ARMS primers for miR128a rs11888095 C/T				
miR128 Fo	5-AGTATGGAATTTTTACTGTGTTGTCTGT-3	Control band	441 bp	55 °C
miR128 Ro	5-GCCAATTATTGCAAAATATTAAATGTATATGG-3			
miR128 FI	5-ATGTATGCTTTGAATACTGTGAAGGAT-3	T-allele	202 bp	
miR128 RI	5-ATACTATACCACACTCCTTATATGCATTG-3	C-allele	295 bp	
Primers for VEGF -2549 insertion/deletion (I/D) gene polymorphism				
VEGF F	5′-GCTGAGAGTGGGGCTGACTAGGTA-3′	D-allele	211 bp	58.8 °C
VEGF R	5′-GTTTCTGACCTGGCTATTTCCAGG-3′	I-allele	229 bp	
Primer sequence of ACE I/D rs4646994				
ACE F	5′- GTGGAGACCACTCCCATCCTTTCT -3′	D	190bp	58 °C
ACE R	5′- GATGTGGCCATCAACTTCGTCACGAT -3′	I	490bp	

**Table 2:** The distribution of *VEGF* rs699947 C/A genotypes in T2D cases and controls.

Subjects	*n*	CC	%	CA	%	AA	%	Df	X2	C	A	*p*-Value
Cases	122	42	34.4	70	57.4	10	8.2	2	9.93	0.63	0.37	0.007
Controls	126	58	46	48	38.1	20	15.9			0.60	0.40	

**Table 3:** The distribution of *VEGF*-2549 I/D polymorphism genotypes in T2D cases and controls.

Subjects	*n*	I	%	ID	%	D	%	Df	X2	I	D	*p*-Value
Cases	133	20	15	71	53.4	42	31.6	2	6	0.42	0.58	0.049
Controls	133	30	22.6	77	57.9	26	19.5			0.52	0.48	

**Table 4:** Genotype distribution of *ACE* I/D rs4646994 between T2D cases and controls.

Subjects	*n*	II %	ID %	DD %	I	D	Df	X2	*p*-Value
Cases	152	87 (57.23%)	55 (36.2%)	10 (6.57%)	0.75	0.25	2	92.43	<0.0001
Controls	150	18 (12%)	60 (40%)	72 (48%)	0.32	0.68			

**Table 5:** Genotype distribution *mir128* rs11888095 C/T genotypes in T2D cases and controls.

Subjects	*n*	CC	CT	TT	C	T	Df	X2	*p*-Value
Cases	129	35 (27.13%)	68 (52.71%)	26 (20.16)	0.54	0.46	2	20.06	0.0001
Controls	112	62 (55.35%)	38 (33.92%)	12 (10.70%)	0.73	0.27			

**Table 6:** Association of *VEGF* rs699947 C/A SNP with T2D.

Genotypes	Healthy Controls		T2D Cases		OR (95% CI)	Risk Ratio (RR)	*p*-Value
	(*n* = 126)	%	(*n* = 122)	%			
Codominant							
VEGF–(C)	58	46	42	34.4	1 (ref.)	1 (ref.)	
VEGF-(CA)	48	38.1	70	57.4	2.01 (1.17–3.45)	1.42 (1.08–1.87)	0.011 *
VEGF-(A)	20	15.9	10	8.2	0.69 (0.29–1.62)	0.87 (0.64–1.17)	0.363
Dominant							
VEGF (C)	58	46	42	34.4	1 (ref.)	1 (ref.)	
VEGF-(CA + A)	68	54	80	65.6	1.62 (0.97–2.71)	1.26 (0.99–1.60)	0.05
Recessive							
VEGF-(C + CA)	106	84	112	91.8	1 (ref.)	1 (ref.)	
VEGF–(A)	20	16	10	8.2	0.47 (0.211–1.05)	0.72 (0.54–0.97)	0.06
Allele							
VEGF-(C)	164		154		1 (ref.)	1 (ref.)	
VEGF-(A)	88		90		1.08 (0.75–1.57)	1.04 (0.86–1.25)	0.64

* Statistically significant difference.

**Table 7:** Association of *VEGF* rs699947 C/A genotypes with T2D patients’ characteristics.

Clinical Feature			CC 42	CA 70	AA 10	X2	DF	*p*-Value
Gender								
	Male	82	18 (14.75%)	58 (47.54%)	6 (4.91%)	19.32	2	0.0001 *
	Female	40	24 (19.67%)	12 (9.83%)	4 (3.27%)			
Age								
	>40	91	36 (29.50%)	50 (40.98%)	5 (4.09%)	6.3	2	0.042 *
	>25	31	6 (4.8%)	20 (9.6%)	5 (1.9%)			
HbA1c%								
	>6	102	36 (29.50%)	60 (49.18%)	6 (4.91%)	4.43	2	0.100
	<6	20	6 (4.91%)	10 (8.19%)	4 (3.27%)			
TG mg/dl								
	<200	49	25 (51%)	20 (40.81%)	4 (8.16%)	10.16	2	0.005 *
	>200	73	17 (23.28%)	50 (68.49%)	6 (8.21%)			
TC mg/dl								
	<200	64	12 (18.75%)	45 (70.31%)	7 (10.93%)	14.12	2	0.006 *
	>200	58	30 (51.72%)	25 (43.10%)	3 (5.17%)			
LDL-C mg/dl								
	<100	66	32 (57.57%)	30 (45.45%)	4 (6.06%)	12.6	2	0.0018 *
	>100	56	10 (10.85%)	40 (71.42%)	6 (10.71%)			
HDL-C mg/dl								
	<55	49	9 (18.36%)	34 (69.38%)	6 (12.24%)	8.32	2	0.015 *
	>55	73	31 (42.46%)	38 (52%)	4 (5.47%)			
VITD ng/mL								
	<30	16	6 (5.8%)	10 (9.6%)	0 (0%)	1.343	4	0.854
	>30	14	5 (4.8%)	8 (7.7%)	1 (1%)			

The association of *VEGF* rs699947 C/A genotypes with T2D patients characteristics. The gender, age, HbA1c%, cholesterol mg/dl, LDL-C mg/dl and HDL-C mg/dl are based on data for 122 cases. For the vitamin D ng/mL (VITD), data was collected for 30 cases. * Statistically significant difference.

**Table 8:** Association of *VEGF*-2549 I/D polymorphism with T2D.

Genotypes	Healthy Controls		T2D Cases		OR (95% CI)	Risk Ratio (RR)	*p*-Value
	(*n* = 133)	%	(*n* = 133)	%			
Codominant							
VEGF–(I)	30	22.55	20	15.03	1 (ref.)	1 (ref.)	
VEGF-(ID)	77	57.89	71	53.38	1.38 (0.72–2.65)	1.15 (0.87–1.51)	0.32
VEGF-(D)	26	19.54	42	31.57	2.42 (1.14–5.11)	1.56 (1.07–2.28)	0.010 *
Dominant							
VEGF (I)	30	22.55	20	15.03	1 (ref.)	1 (ref.)	
VEGF-(ID + D)	103	77.44	113	84.96	1.64 (0.88–3.07)	1.25 (0.96–1.64)	0.090
Recessive							
VEGF-(I + ID)	107	80.45	91	68.42	1 (ref.)	1 (ref.)	
VEGF–(D)	26	19.54	42	31.57	1.89 (1.08–3.33)	1.37 (0. 98–1.91)	0.025 *
Allele							
VEGF-(I)	133	100	111	83.45	1 (ref.)	1 (ref.)	
VEGF-(D)	129	97	155	116.54	1.43 (1.02–2.03)	1.01 (0.83–1.21)	0.037 *

* Statistically significant difference.

**Table 9:** Association of *VEGF*-2549 I/D genotypes with T2D patients’ characteristics.

Clinical Feature	*n*	D %	I %	ID %	X2	DF	*p*-Value
Gender							
Female	40	7 (6.5%)	9 (8.4%)	24 (22.4%)	1.498	2	0.473
Male	67	14 (13.1%)	9 (8.4%)	44 (41.1%)			
Age							
>25	16	4 (3.7%)	1 (0.9%)	11 (10.3%)	1.607	2	0.448
>40	91	17 (15.9%)	17 (15.9%)	57 (53.3%)			
HbA1c%							
<6	1	0 (0.0%)	0 (0.0%)	1 (0.9%)	0.579	2	0.749
>6	96	21 (19.6%)	18 (16.8%)	67 (62.6%)			
TC mg/dl							
<200	65	12 (11.2%)	7 (6.5%)	46 (43.0%)	11.411	4	0.022 *
>200	17	6 (35.29%)	2 (11.76%)	9 (52.94%)			
TG mg/dl							
<200	65	12 (18.46%)	7 (10.76%)	46 (70.76%)	11.0	2	0.004 *
>200	31	12 (38.70%)	8 (16.12%)	11 (35.48%)			
LDL-C mg/dl							
<100	34	4 (3.7%)	2 (1.9%)	26 (24.3%)	11.364	4	0.023 *
>100	51	14 (13.1%)	8 (7.5%)	29 (27.1%)			
HDL-C mg/dl							
<55	26	17 (15.9%)	9 (8.4%)	45 (42.1%)	13.106	4	0.011 *
>55	12	1 (0.9%)	0 (0.0%)	11 (10.3%)			
VIT D ng/ml							
<30	22	3 (2.8%)	2 (1.9%)	17 (15.9%)	2.777	4	0.596
>30	15	3 (2.8%)	2 (1.9%)	10 (9.3%)			

* Statistically significant difference. Association of VEGF-2549 I/D Genotypes with T2D Patient’s Characteristics.

**Table 10:** Association of *ACE* I/D polymorphism with T2D.

Genotypes	Healthy Controls	T2D Cases	OR (95% CI)	Risk Ratio (RR)	*p*-Value
	(*n* = 150)	(*n* = 152)			
Co-dominant					
ACE–II	18	87	1 (ref.)	1 (ref.)	
ACE–ID	60	55	0.18 (0.1–0.35)	0.32 (0.208–0.518)	0.0001 *
ACE–DD	72	10	0.128 (0.01–0.1)	0.19 (0.1272–0.2996)	<0.0001 *
Dominant					
ACE–II	18	87	1 (ref.)	1 (ref.)	
ACE–(DI+DD)	132	65	0.10 (0.06–0.18)	0.25 (0.1661–0.3940)	<0.0001 *
Recessive					
ACE–(II+DI)	78	142	1 (ref.)	1 (ref.)	
ACE–DD	72	10	0.076 (0.04–0.15)	0.40 (0.3–0.49)	<0.0001 *
Allele					
ACE–I	96	229	1 (ref.)	1 (ref.)	
ACE–D	204	75	0.10 (0.1–0.2)	0.40 (0.33–0.48)	0.0001 *
Over dominant					
ACE–II+DD	90	97	1 (ref.)	1 (ref.)	
ACE–ID	60	55	0.85 (0.5–1.4)	0.92 (0.73–1.16)	0.4910

* Statistically significant difference.

**Table 11:** Association of *ACE* I/D polymorphism genotype with patients’ characteristics.

Clinical Feature	*n*	*n* = 152	II	DI	DD	X2	DF	*p*-Value
Gender								
	Male	44	25	17	02	0.49	2	0.78
	Female	108	62	38	08			
Age								
	>25	96	41	46	09	22.62	2	0.0001 *
	<25	56	46	09	01			
HBA1c%								
	>6	103	52	45	06	7.79	2	0.020 *
	<6	49	35	10	04			
TG mg/dl								
	<200	80	46	25	09	6.74	2	0.0344 *
	>200	72	41	30	01			
TC mg/dl								
	<200	99	50	43	06	6.49	2	0.039 *
	>200	53	37	12	4			
LDL-C mg/dl								
	<100	82	37	30	01	7.19	2	0.022 *
	>100	70	50	25	09			
HDL-C mg/dl								
	<55	104	58	42	04	5.47	2	0.64
	>55	48	29	13	06			

* Statistically significant difference.

**Table 12:** Association of *miR128* rs11888095 C/T with T2D.

Genotypes	Healthy Controls	T2D Cases	OR (95% CI)	Risk Ratio (RR)	*p*-Value
	(*n*)	(*n*)			
Codominant					
miR128 –(C)	62	35	Ref	Ref	
miR128-(CT)	38	68	3.16(1.8–5.6)	1.78(1.3–2.4)	0.0001 *
miR128-(T)	12	26	3.83(1.7–8.5)	2.0 (1.2–3.3)	0.0010 *
Dominant					
miR128-(C)	62	35	Ref	Ref	
miR128-(CT + T)	50	94	3.3(1.9–5.7)	1.84(1.4063–2.4097)	<0.0001 *
Recessive					
miR128-(C + CT)	100	103	Ref	Ref	
miR128–(T)	12	26	3.3(1.9–5.7)	1.55(0.96–2.5)	<0.0001 *
Allele					
miR128-(C)	112	129	Ref	Ref	
miR128-(T)	62	120	1.68(1.13–2.5)	1.36 (1.1–1.7)	0.0105 *

* Statistically significant difference.

**Table 13:** Association of *miR128* rs11888095 C/T genotypes with T2D patients’ characteristics.

Clinical Feature	*n*		CC	CT	TT	X2	DF	*p*-Value
Gender		129	35	68	26			
	Male	40	7	28	05	6.95	2	0.031 *
	Female	89	28	40	21			
Age								
	>25	109	26	59	25	7.53	2	0.021 *
	<25	20	10	09	01			
HBA1c%								
	>6	82	12	49	21	18	2	0.0001 *
	<6	47	23	19	05			
TG mg/dl								
	<200	82	14	48	20	11.84	2	0.0027 *
	>200	47	21	20	6			
TC mg/dl								
	<200	85	28	40	17	0.52	2	0.776
	>200	44	17	18	9			
LDL-C mg/dl								
	<100	56	08	34	14	8.37	2	0.0152 *
	>100	73	27	34	12			
HDL-C mg/dl								
	<55	98	25	54	19	0.96	2	0.6188
	>55	31	10	14	07			

* Statistically significant difference.

## Data Availability

Not applicable.
